# A survey of the usage (frequency and pattern) of antibiotics at the University of Maiduguri Veterinary Teaching Hospital, Maiduguri, Borno State, Nigeria

**DOI:** 10.14202/vetworld.2021.2941-2946

**Published:** 2021-11-24

**Authors:** Nubwa Daniel, Kefas David Malgwi, Bukar Umaru, Isaac John Omeh, Ladi Sanya

**Affiliations:** 1 Department of Veterinary Pharmacology and Toxicology, Faculty of Veterinary Medicine, University of Maiduguri, Maiduguri, Borno State, Nigeria; 2 Veterinary Teaching Hospital, University of Maiduguri, Maiduguri, Borno State, Nigeria; 3 Department of Veterinary Physiology and Biochemistry, Faculty of Veterinary Medicine, University of Maiduguri, Maiduguri, Borno State, Nigeria

**Keywords:** antibiotics, frequency, pattern, survey, usage

## Abstract

**Background and Aim:**

Veterinary antibiotics are widely used to treat bacterial diseases in various species of animals. However, despite the importance of these chemotherapeutic agents, their indiscriminate or extensive use can pose dangers to the animals or humans that consume edible tissues from animals contaminated with antibiotic residues. Therefore, concerns regarding their appropriate and judicious use in animals are of public health significance. This is because of the tendencies of developing resistance to targeted microbes and the ability of the parent compound or its metabolites to persist as residues in the animal tissues. This study aimed to determine the frequency and pattern of antibiotic usage and ascertain the level of awareness of clinicians on the judicious use of antibiotics at the University of Maiduguri Veterinary Teaching Hospital, Maiduguri, Borno State, Nigeria.

**Materials and Methods:**

Data related to the administration of antibiotics in all species of animals presented for treatment from January 2009 to December 2018 were obtained from the hospital archives, with permission from the office of the hospital directorate. The diseases, hospital units, antibiotics used, and years were parameters that were recorded for each case. Furthermore, 47 questionnaires were administered to clinicians who render services to the hospital.

**Results:**

A total of 63.9% of all cases brought to the hospital within the 10 years under study were infectious, and as such, were treated with antibiotics. The highest recorded use of antibiotics was observed in the poultry unit (38.4%), followed by the large animal unit (24.1%), with the lowest used recorded in the ambulatory unit (9.3%). Furthermore, regarding the antibiotics used, oxytetracycline had the highest occurrence (55%), followed by penicillin-streptomycin combination (12.2%), with the lowest being metronidazole (0.30%). The highest number of cases treated with antibiotics was recorded in 2018 (22.5%), and the lowest was recorded in 2014 (1.3%). Regarding the questionnaire administered to the clinicians, 78.7% of the respondents preferred oxytetracycline as their drug of choice, whereas only 2.1%, 4.3%, 2.1%, and 4.3% preferred amoxicillin, penicillin, streptomycin, and penicillin-streptomycin, respectively. Moreover, 65.9% of the respondents used a particular antibiotic because of its availability at the hospital, 8.5% because of cost, and 27.7% because of clinician preference. Furthermore, 74.5% of the clinicians offered palliative intervention while awaiting laboratory reports, whereas 8.5% treated the animals without requesting laboratory analyses.

**Conclusion:**

In this study, oxytetracycline was found to be the most used antibiotic for treating infectious diseases at the hospital because of its availability. The observed pattern appeared in the following order of frequency: Oxytetracycline, penicillin-streptomycin combination, neomycin, erythromycin, amoxicillin, tylosin, streptomycin, and gentamicin with metronidazole being the least frequent. There might also be antibiotic resistance, which requires a change to another antibiotic because of the lack of response to the initial antibiotic. Non-judicious antibiotic use can also have a negative impact on public health because of the development of multidrug-resistant “superbugs” and the problem of drug residue.

## Introduction

Antibiotics are compounds that are synthesized by various microorganisms (bacteria, fungi, and actinomycetes) and may kill or inhibit multiplying bacteria rapidly [1]. An antibiotic can be classified as having either a broad or narrow spectrum activity. A broad-spectrum antibiotic is active against Gram-positive and Gram-negative bacteria, as well as larger viruses, protozoa, etc. In contrast, a narrow-spectrum antibiotic acts on a relatively small number of microorganisms [2,3]. Antibiotics are commonly used in veterinary medicine to treat infectious diseases caused by bacteria and other microorganisms. They are also used as growth promoters in food animals [4].

The worldwide increase in the use of ­antibiotics as an integral part of livestock and poultry production to treat and prevent infectious diseases and as growth promoter has led to the development of antibiotic resistance over the years [5,6]. When resistance occurs, previously effective drugs can no longer be considered as such, and new drugs must be developed [7]. Resistance may develop in several ways, such as improper use of antibiotics for growth promotion, indiscriminate use of antibiotics without a prescription from professionals, and under-dose usage of antibiotics [8]. Similarly, non-judicious use of antibiotics can also lead to the problem of drug residues, which can be found in the edible tissues of animals and animal products and pose great dangers to human health when consumed. However, when used properly (i.e., the right antibiotic is used and is given as prescribed for the right amount of time), antibiotics are less likely to contribute to the development of antibiotic-resistant organisms [8]. Guidelines on the responsible and prudent use of antibiotics in animal husbandry were issued by the United Nations Organization of UN-Office International des Epizooties [9] and confirmed by the European Union [10]. These guidelines aim to maintain antibiotic efficacy, avoid dissemination of resistant bacteria, and, finally, avoid such bacteria from reaching human food. Antibiotics should be applied and directed during the different steps of treatment, from prescription up to the withdrawal period, under the supervision of professionals. Practices indicated a need to improve sensitivity testing services and facilities before prescribing a specific antibiotic [11].

This study aimed to determine the frequency and pattern of antibiotic usage and to ascertain the level of awareness of clinicians on the judicious use of antibiotics at the University of Maiduguri Veterinary Teaching Hospital (UMVTH), Maiduguri, Borno State, Nigeria.

## Materials and Methods

### Ethical approval

This study was approved by the Directorate of the Veterinary Teaching Hospital, University of Maiduguri, Borno State, Nigeria. The consent of the veterinarians working at the hospital was also obtained before the questionnaires were administered.

### Study period and location

This study was achieved by obtaining relevant data from the hospital archives related to the administration of antibiotics in all species of animals that were presented for treatment from January 2009 to December 2018. The disease, units, antibiotics used, and years were parameters that were recorded for each case. Furthermore, 47 questionnaires were administered to clinicians who render services to the hospital. The survey was carried out at the UMVTH, Maiduguri, Borno State, Nigeria. Established in 1983, the UMVTH is the only veterinary teaching hospital in the North-Eastern area of Nigeria. It sometimes partners with national and international ­organizations because of its outstanding performance. It also receives referrals from public veterinary hospitals, private veterinarians, and clinics.

### Statistical analysis

The data generated were analyzed based on descriptive statistics using Microsoft Office Excel^®^ 2013 (Microsoft Corporation, NY, USA).

## Results

From the survey performed here, a total of 1,580 cases were documented in the UMVTH over the 10 years under study, with 1010 of the cases being infectious and, as such, being treated with antibiotics; that is, antibiotics were administered to 63.9% of all cases brought to the hospital. The highest use of antibiotics (Table-1) was recorded in the poultry unit (38.4%), followed by the large animal unit (24.1%), with the lowest use observed in the ambulatory unit (9.3%). Moreover, regarding the antibiotics used, oxytetracycline was administered most frequently (55%), followed by penicillin-streptomycin combination (12.2%), with least frequent being metronidazole (0.30%). The highest number of cases treated with antibiotics was recorded in 2018 (22.7%), and the lowest was recorded in 2014 (1.3%).

**Table-1:** Frequency of use of each drug in each unit of the UMVTH.

S. No.	Drugs	Large animal	Small animal	Ambulatory	Surgery	Avian
1.	Oxytetracycline	183	97	59	49	167
2.	Pen-strep	16	16	22	65	4
3.	Tylosin	16	1	2	1	11
4.	Procaine-penicillin	6	6	2	3	1
5.	Metronidazole	2	1	-	-	-
6.	Amoxicillin	16	7	-	6	7
7.	Penicillin	1	-	-	-	4
8.	Enrofloxacin	2	3	-	-	4
9.	Gentamicin	-	14	2	-	6
10.	Ceftriaxone	-	2	2	7	-
11.	Ciprofloxacin	-	-	-	9	6
12.	Streptomycin	-	3	1	-	25
13.	Neomycin	1	-	3	1	75
14.	Doxycycline	-	-	1	-	12
15.	Erythromycin	-	-	-	-	58
16.	Florfenicol	-	-	-	-	8
	Total	243	150	94	135	388
	Percentage	24.06	14.85	9.31	13.37	38.42

UMVTH=University of Maiduguri Veterinary Teaching Hospital

The questionnaire administered to the clinicians revealed that 78.7% of the respondents preferred oxytetracycline as their drug of choice, whereas only 2.1%, 4.3%, 2.1%, and 4.3% preferred amoxicillin, penicillin, streptomycin, and penicillin-streptomycin, respectively. Moreover, 65.9% of the respondents used a particular antibiotic because of its availability at the hospital, whereas 27.7% of the antibiotic usage was based on clinicians’ preferences. In addition, only 4.3% of the clinicians reported attending a seminar on the prudent use of antibiotics and rational approach to prescription organized by the UMVTH. Furthermore, 42.6% of the clinicians were aware of the guidelines of the Veterinary Council Nigeria (VCN) on the judicious use of antibiotics in veterinary hospitals, whereas 57.4% of them were unaware of them. The questionnaire also showed that 53.2% of the respondents had to change their line of treatment because there was no response to the initial treatment. This might have been caused by possible resistance of the offending microbe to the antibiotic being used. The records revealed that there was a remarkable positive outcome after changing the line of treatment to another antibiotic. The highest recorded antibiotic switch was from oxytetracycline to other antibiotics, such as ceftriaxone, streptomycin, and cotrimoxazole. Moreover, 74.5% of the clinicians provided supportive treatment while waiting for laboratory reports, whereas 8.5% treated the animals without requesting laboratory analyses. The questionnaire showed that 40.4% of the respondents who sent samples to the laboratory did not request a sensitivity test for the isolated organism before instituting antibiotic therapy.

## Discussion

**Figure-1:**
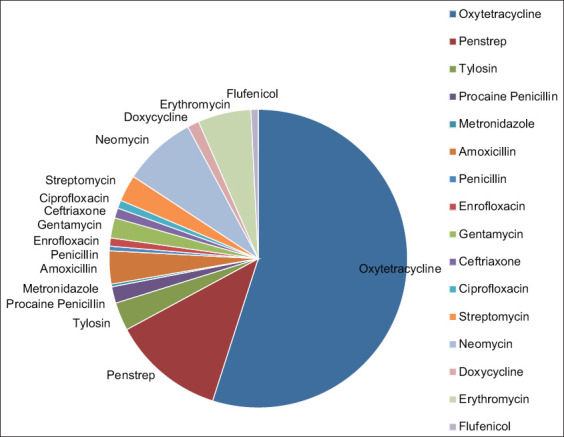
Frequency of antibiotics used in the University of Maiduguri Veterinary Teaching Hospital. Penstrep=Penicillin +streptomycin.

From the recorded cases that were presented to the UMVTH during the period under study, about two-thirds of the cases were infectious and were, therefore, treated with antibiotics. Other cases were non-infectious, such as poisoning, metabolic disorders, and fractures. The prevalent usage of antibiotics (i.e., oxytetracycline, penicillin-streptomycin, neomycin, erythromycin, etc., in that order) was largely based on the frequency of use rather than the volume constituting the dose of the drug. Oxytetracycline was mostly used in large animals, followed by poultry and small animals (Table-1). This might be because the large animal and poultry units are the most visited and busy units than the small animal units (Table-2), possibly because of environmental and cultural factors. Table-3 and Figure-1 show higher usage of oxytetracycline (78.7%) by clinicians, which might not be unconnected to its availability, affordability, and broad-spectrum activity, because most treatments (74.5%) at the hospital were carried out based on clinical signs. This agrees with the findings of Aliyu *et al*. [12], who reported that only 2% of animal health practitioners in Niger state, Nigeria, request laboratory results before instituting treatment. Different researchers have reported oxytetracycline as being the drug most widely used by farmers, para veterinarians, and veterinarians. Geidam *et al*. [13] reported that oxytetracycline (36.5%) was the antibiotic most commonly marketed and used by poultry farmers, even without a prescription, in Maiduguri. Aliyu *et al*. [12] reported that the percentage of use of antibiotics by animal health practitioners (veterinarians and para veterinarians) is 99.2% for oxytetracycline, which is the highest in Niger state. The use of gentamicin has been banned in food-producing animals because of its higher tendency to remain in food as residues. In this study, however, the use of gentamicin (Table-1) was restricted to small animals and, to a small extent, poultry (because of incorporation with other drugs). This is a good indication that clinicians are aware of this fact and use it only in companion animals.

**Table-2:** Percentage of use of antibiotics in each unit of the UMVTH.

S. No.	Units	Percentage
1.	Large animal	24.1
2.	Small animal	15.1
3.	Ambulatory	9.3
4.	Surgery	13.2
5.	Avian	38.4
	Total	100.0

UMVTH=University of Maiduguri Veterinary Teaching Hospital

**Table-3:** Response to a questionnaire survey on the use of antibiotics by clinicians at the UMVTH

Survey questions	Respondents	Respondents (%)
Which antibiotic do you prefer or frequently use in the UMVTH?		
Oxytetracycline	37	78.7
Amoxicillin	1	2.1
Penicillin		4.3
Streptomycin	1	2.1
Pen-strep		4.3
Others		8.5
What are your reasons for the choice of any antibiotic in the UMVTH?		
Availability	31	66
Cost	0	0
Clinician preference	13	27.7
Others	3	6.4
Has there ever been training or seminar exclusively by the UMVTH to its clinicians on the prudent use of antibiotics and rational approach to prescription?		
Yes	2	4.3
No	45	95.7
Do you know if there are any guidelines by the VCN on the judicious use of antibiotics in veterinary hospitals and clinics?		
Yes	20	42.6
No	27	57.4
Has there been resistance to any antibiotic you used to the point that you have to switch your line of treatment to another antibiotic?		
Yes	25	53.2
No	22	46.8
Do you treat symptomatically, awaiting laboratory results or before instituting any antibiotic, or do you administer antibiotics without any laboratory findings?		
Symptomatically awaiting laboratory result	35	74.5
Without requesting for laboratory result	4	8.5
I do both	8	17.0
Do you demand sensitivity test after confirmatory diagnosis before instituting antibiotic therapy?		
Yes	19	40.4
No	28	59.6

UMVTH=University of Maiduguri Veterinary Teaching Hospital

Furthermore, from this survey, it was observed that (Table-3) most clinicians used a particular antibiotic because of its availability (66%), rather than preference (27.7%) or cost (8.5%). Therefore, the availability of other antibiotics (especially narrow-spectrum ones) may help clinicians choose drugs for the treatment of infectious diseases.

Most veterinary national and international conferences are now focused on drug resistance and residues because it has become a global concern for human and animal health. The recent Nigerian Veterinary Medical Association conventions and VCN continuing education (2016-2018) have been focused mostly on this global epidemic. A list of about 200 essential veterinary drugs, pesticide chemicals, and biologicals has been published by the VCN and referred to as the Nigerian Veterinary Formulary. Moreover, Aliu [14] reported in his paper presentation that this list should be reviewed at least once every 5 years and whenever necessary. Furthermore, new drugs should be introduced only if they offer a distinct advantage over previously selected drugs. If new information becomes available on drugs already on the list, this clearly shows they no longer have a favorable risk-benefit ratio. In that case, they should be deleted and replaced with a safer drug.

Table-4 shows an increase in the cases that were presented at the hospital, with the highest number recorded in 2018, which might be attributed to an increase in the awareness of the need to report cases of sick animals to the hospital. It might also be connected to the house-to-house campaign implemented by the management of the UMVTH in 2017 within the university community. There was also a decrease in the cases presented to the hospital in 2014, which may either have been caused by the height of the insurgency or inadequate record taking that occurred during that year.

**Table-4:** Yearly distribution of antibiotics used in the UMVTH.

S. No.	Drugs	2009	2010	2011	2012	2013	2014	2015	2016	2017	2018
1.	Oxytetracycline	80	69	31	23	36	4	16	84	97	115
2.	Penicillin-streptomycin	4	9	13	13	30	4	7	17	22	4
3.	Tylosin	1	5	1	2	2	-	2	15	1	2
4.	Procaine-penicillin	3	2	2	-	-	-	2	5	1	3
5.	Metronidazole	-	-	-	-	-	-	-	2	1	-
6.	Amoxicillin	-	-	-	-	-	-	-	3	5	28
7.	Penicillin	-	1	-	-	-	-	-	1	-	3
8.	Enrofloxacin	-	1	2	-	-	1	-	3	2	-
9.	Gentamicin	2	6	-	-	-	3	-	6	1	4
10.	Ceftriaxone	-	-	-	-	4	1	-	4	-	2
11.	Ciprofloxacin	-	-	-	-	-	-	-	1	2	6
12.	Streptomycin	3	-	1	-	-	-	-	7	5	13
13.	Neomycin	-	-	-	-	7	-	6	26	18	23
14.	Doxycycline	-	-	-	-	2	-	1	6	1	3
15.	Erythromycin	-	-	-	-	-	-	4	18	15	21
16.	Florfenicol	-	-	-	-	5	-	-	1	-	2
	Total	93	93	50	38	86	13	38	199	171	229
	Percentage	9.21	9.21	4.95	3.76	8.51	1.29	3.76	19.70	16.93	22.67

UMVTH=University of Maiduguri Veterinary Teaching Hospital

## Conclusion

The findings of this study revealed that ­oxytetracycline was the most used antibiotic at the UMVTH, Maiduguri, Borno State, Nigeria, followed by a penicillin-streptomycin combination (Table-5). This was mostly due to its constant availability at the hospital. The study also revealed some resistance to a particular antibiotic (especially oxytetracycline) experienced by clinicians, which prompted them to change their treatment line to another antibiotic, which yielded good results. It was also observed that a more significant percentage of the clinicians treated their patients symptomatically as they awaited laboratory results.

**Table-5:** Frequency and percentage of use of antibiotics in the UMVTH.

S. No.	Drugs	Frequency	Percentage
1.	Oxytetracycline	555	54.95
2.	Penicillin-streptomycin	123	12.18
3.	Tylosin	31	3.07
4.	Procaine penicillin	18	1.78
5.	Metronidazole	3	0.30
6.	Amoxicillin	36	3.56
7.	Penicillin	5	0.50
8.	Enrofloxacin	9	0.89
9.	Gentamicin	22	2.18
10.	Ceftriaxone	11	1.09
11.	Ciprofloxacin	9	0.89
12.	Streptomycin	29	2.87
13.	Neomycin	80	7.92
14.	Doxycycline	13	1.29
15.	Erythromycin	58	5.74
16.	Florfenicol	8	0.79
	Total	1010	100.00

UMVTH=University of Maiduguri Veterinary Teaching Hospital

## Authors’ Contributions

ND and KDM: Designed the study, wrote the manuscript, and prepared tables and figures. BU: Supervised the study, reviewed, and edited the manuscript. IJO and LS: Assisted in the issuance and compilation of the questionnaires and statistical analysis. All authors read and approved the final manuscript.

## References

[1] Matthew I.H, Andrew W.T, Barrie W (2019). Antibiotics:Past, present, and future. Curr. Opin. Microbiol.

[2] Adzitey F (2015). Antibiotic classes and antibiotic susceptibility of bacterial isolates from selected poultry, a mini-review. Worlds Vet. J.

[3] Eagar H, Swan G, Van Vuuren M (2012). A survey of antimicrobial usage in animals in South Africa with specific reference to food animals. J. S. Afr. Vet. Assoc.

[4] Russell J.B, Jarvis G.N (2008). Practical mechanisms for interrupting the Oral-fecal lifecycle of *Escherichia coli*. J. Mol. Microbiol. Biote chnol.

[5] Collignon P (2003). A review-the use of antibiotics in food production animals-does this cause problem in human health. Manipulating pig production IX. In:Proceedings of the Ninth Biennial Conference of the Australasian Pig Science Association (Inc.) (APSA), Fremantle, Western Australia, 23-26 November.

[6] Apata D.F (2009). Antibiotic resistance in poultry. Int. J. Poult. Sci.

[7] Nisha A.R (2008). Antibiotic residues-a global health hazard. Vet. World.

[8] Padol A.R, Malapure C.D, Domple V.D, Kamdi B.P (2015). Occurrence, public health implications and detection of antibacterial drug residues in raw milk. Environ. We Int. J. Sci. Technol.

[9] OIE (2013). OIE Global Conference on the Responsible and Prudent Use of Antimicrobial Agents for Animals:International Solidarity to Fight against Antimicrobial Resistance, Paris.

[10] EU. Commission Notice. Guidelines for the prudent use of antimicrobials in veterinary medicine (2015). Official J. Eur. Union.

[11] Magdy M.E.S, Mohamed B.M.A (2017). Necessary Usage of Antibiotics in Animals. Department of Food Toxins and Contaminants, National Research Center, Cairo, Egypt.

[12] Aliyu E.H, Nma B.A, Suleiman Y, Muhammed K.Y (2018). A survey of antimicrobial usage by animal health practitioners in Niger State. In:A Paper Presentation in the 55^th^ Annual Congress of the Nigerian Veterinary Medical Association. Animal Production and Public Health Oral Presentations.

[13] Geidam Y.A, Ibrahim U.I, Grema H.A, Sanda K.A, Suleiman A, Mohzo D.L (2012). Patterns of antibiotic sales by drug stores and usage in poultry farms:A questionnaire-based survey in Maiduguri, Northeastern Nigeria. J. Anim. Vet. Adv.

[14] Aliu Y.O (2016). Drug use, misuse and residue:Implication for economic diversification. In:A Paper Presented at the VCN Professional Continuing Education Seminar, Niger State, Nigeria.

